# Investigation of Selected Polymer Composite-Aluminum Oxide Coating Tribological Systems

**DOI:** 10.3390/ma13235491

**Published:** 2020-12-02

**Authors:** Joanna Korzekwa, Elżbieta Bociąga, Dariusz Bochenek

**Affiliations:** Institute of Materials Engineering, Faculty of Science and Technology, University of Silesia in Katowice, 75 Pułku Piechoty 1a, 41-500 Chorzów, Poland; elzbieta.bociaga@us.edu.pl (E.B.); dariusz.bochenek@us.edu.pl (D.B.)

**Keywords:** adhesion contact, coating, coefficient of friction, polymer composite, surface roughness

## Abstract

The tribotesting of friction systems requires discussion on proper selection of its conditions and data presentation. System tribology is based, for example, on analysis of the friction contact, the roughness of the cooperating surfaces, and the wear rate of the rubbing elements or coefficient of friction in relation to the sliding distance. Friction pairs, consisting of an aluminum alloy sample with an oxide layer (Al_2_O_3_) with and without the addition of inorganic fullerenes like tungsten disulphide (IF-WS_2_) nanoparticles on its surface cooperating with a counter-sample made of polymer composites prepared on the basis of phenol-formaldehyde resin with different fillers, were tested using a device with a pin-on-plate friction pair system. The results of the experiments showed sufficient durability of the Al_2_O_3_ and Al_2_O_3_/IF-WS_2_ oxide coatings in combination with the polymer composite. It was found that resin fillers such as cotton fibers, jute fibers, molybdenum disulphide (MoS_2_) or graphite (C) influence the friction behavior of the tribological pairs. Although the values of the coefficient of friction obtained in the tests were quite high, their course during the tests ensured stable cooperation of the aluminum coating/polymer composite friction pair on a 15 km distance, under a load of 0.5 MPa. The lowest coefficients of friction were obtained for oxide layers formed on aluminum combined with a polymer composite filled with cotton fibers and graphite. These studies provide information on the tribological properties of commercially available polymer composites cooperating with the produced oxide coatings, supplementing the available literature with the results of research on new, so far unexplored tribological partners. Microscopic investigation of the structure and morphology of the formed surface oxide layers and also microgeometry studies of both the friction elements were used to better understand the obtained research results.

## 1. Introduction

Aluminum alloys are highly valued construction materials because of their good corrosion resistance, high impact strength at low temperatures and favorable strength-to-density ratio. These properties make aluminum alloys a competitive material for steel and cast iron in the automotive and aerospace industries, where vehicle weight affects the efficiency of vehicle use, and, thus, the environment [1]. Due to their excessive tendency to form adhesive junctions, the aluminum alloys are not suitable to be directly applied as the cooperating parts of machinery and equipment. One of the ways to eliminate the adverse effects of adhesion on the aluminum surfaces of machine components is an anodizing method widely used both in industry and in laboratory studies [2]. An anodic aluminum oxide (AAO) coating made on the substrate of aluminum alloys is particularly useful as a protective coating of machine elements [3]. The applications of AAO require appropriate structural properties, which can be achieved through the judicious choice of the chemical and structural modification steps [4]. Its wide range of application is due to its morphological properties, which depend on the substrate preparation [5], anodization voltage, kind of electrolyte [6,7], temperature of the electrolyte [8,9,10], and kind of alloy [11,12]. In recent years, many studies have been devoted to the study of AAO surface layer modification [13,14,15,16,17,18]. The dynamic development of industry aims to develop more efficient materials for use in difficult and complex conditions. The friction and wear processes of materials are unusually complex; as numerous factors influence them. Therefore, the conducting of the experimental research in the field of the production of materials, description of their comprehensive physical–chemical characteristics, and the properties of tribological pairs before introducing them into use is very important for fundamental knowledge. Reducing the friction and wear between interacting surfaces in most tribological applications is usually realized by liquid, lubricants, or solid ones [19,20,21,22,23]. Moreover, surface texturing is one of the known methods of reducing the friction of sliding pairs in the presence of a lubricant [24,25,26,27]. In the friction processes of some materials the phenomenon of the wear is expressed as a transfer of material from one element of the friction pair to another and the transferred material plays the role of a solid lubricant [28,29,30]. That kind of lubrication could be used in many different branches of industry. A solid lubricant can be obtained by methods such as: (a) mixing with oils, a suspension or grease; (b) dry coating as in physical vapor deposition (PVD method), like ion plating or sputtering and as a solid lubricant in a liquid carrier, which is coated (dipping) or sprayed on a solid surface, and then dried; (c) composite materials; and (d) directly applied (rubbed) onto the surface [28]. If additionally, the lubricant is gradually released to the contact surface of the elements of such pairs, then friction and lubrication conditions similar to boundary lubrication could be provided. When a polymer material is rubbing against a harder material, e.g., a metal, polymer particles and also fillers are transferred to the metal counter-sample surface and form a transfer film. The transferred polymer fills the microdefects of the mating surface, reducing its roughness, which results in a lower coefficient of friction and wear. When a transfer film is formed, the polymer part is rubbing against the polymer film on the metal counterpart and not against the metal itself [31,32]. The analysis of the basic tribological properties of selected polymer composites with an Al_2_O_3_ ball and Al_2_O_3_ oxide layers on aluminum alloy was described by [33,34]. In the literature and in industrial practice, data can be found on the modification of polymeric materials by lubricants [35,36]. The best known examples are polyamides modified with graphite, molybdenum disulphide, oil, and solid lubricants. Other well-known examples are layered composites with fillers in the form of long fibers or pieces (sheets, fabrics, and mats), where the matrix is made of curable polymers, e.g., phenol-formaldehyde resin or epoxy resin. Parts of machines and equipment made of such composites are formed by pressing, usually with additional machining, and their production cycle is long.

In tribological issues used in the construction and operation of moving parts of machines, an important task is to select engineering materials constituting the so-called tribological pair, which will improve the efficiency, reliability and durability of various devices. Polymer composites cooperating with elements made of aluminum with an oxide layer are attracting increasing interest due to their application in friction pair as a self-lubricating material. The tribotesting of polymers requires discussion on proper selection of its conditions and data presentation [37].

The presented research problem proposes pairing commercially available polymer composite based on phenol–formaldehyde resin with an Al_2_O_3_/IF-WS_2_ oxide layer that is believed to be a useful solution allowing boundary lubrication in an unlimited time and during the maintenance-free use of a friction pair, e.g., in machines.

## 2. Materials and Methods 

**Figure 1:**
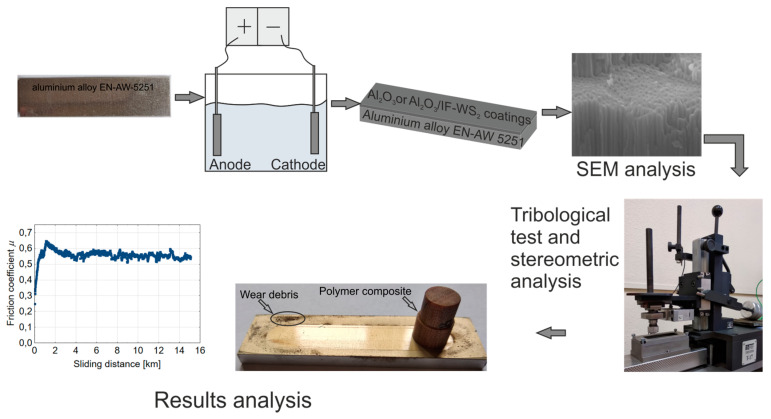
Schematic representation of the study.

The aim of the study was to assess the tribological properties of friction systems: aluminum samples with an oxide layer with and without the addition of tungsten disulphide nanoparticles cooperating with counter-samples made of four composites based on phenol-formaldehyde resin. The friction and wear tests of the samples were carried out with reciprocating movement of the aluminum oxide coatings using a device with a pin-on-plate friction pair system. In tribological tests, the coefficient of friction and the amount of wear of the polymer pin were determined. The surface condition of the friction elements was also analyzed. Schematic representation of the research for this article is shown in Figure 1.

### 2.1. Sample Preparation

#### 2.1.1. Aluminum Oxide Layers

The starting material for the process was EN-AW-5251 aluminum alloy. The samples were cut from a rolled sheet with a roughness determined by parameters: (*Sq*)–0.423 μm, (*Sz*)–2.19 μm, and (*Svk*)–0.453 μm, which was measured by authors of an earlier publication [5]. The samples were etched sequentially with a 5% KOH solution for 45 minutes, and a 10% HNO_3_ solution for 10 minutes, at room temperature. After each step of etching, the sample was placed in distilled water to remove residual acid. The electro-oxidation of the first part of the aluminum alloy samples was carried out in a ternary solution (18% sulfuric (33 mL/L), adipic (67 g/L), and oxalic (30 g/L) acids called the SAS ternary solution). The samples were named Al_2_O_3_-1 to Al_2_O_3_-4. The second part of the samples was obtained in an SAS ternary solution with an admixture of 15 g of a commercially available inorganic fullerene like tungsten disulphide IF-WS_2_ nanoparticles (NanoMaterials Ltd.) per liter of electrolyte. The samples were named Al_2_O_3_/IF-WS_2_-1 to Al_2_O_3_/IF-WS_2_-4. The oxide layers were produced by hard anodizing on the surface of 0.1 dm^2^ of the aluminum alloy. The hard anodizing process was performed using 3 A/dm^2^ current density every half hour. In order to prevent settling of the IF-WS_2_ nanopowder, mechanical stirring was performed every 10 min during the electrolysis process. Table 1 shows the names and descriptions of the studied samples.

#### 2.1.2. Polymer Composite

The samples for testing were obtained from plates with a thickness of approximately 10 mm produced by “IZO-ERG” S.A. in Gliwice (Poland) The plates were formed by a set of preforms pressed at the temperature of 150 °C and at the pressure of 7.85 MPa. Materials with the following compositions were tested: 52% phenol-formaldehyde resin + 48% cotton fabric (named PF cotton), 52.5% phenol-formaldehyde resin + 47.5% jute (named PF + jute), 44.8% phenol formaldehyde resin + 47.5% cotton fabric + 4.7% molybdenum disulphide MoS_2_ (PF+ cotton + MoS_2_), and 44.8% phenol formaldehyde resin + 47.5% cotton fabric + 4.7% graphite C (PF + cotton + C). The cotton fibers were TEX 30 (TEX is the weight in grams of 1000 meters of the thread); jute fibers were TEX 280 and TEX 360 yarns (the weft and warp, respectively); the average grain size of MoS_2_ was 1.15 μm, graphite was the PV 60/65 type. The PV 60/65 determination refers to the degree of fragmentation of the particles and is characterized by testing the residue on a 0.125 mm sieve. For graphite type PV 60/65 this residue is 10%. The cotton fibers and jute used to make the preforms were in the form of sheets. The samples were machined and shaped as a pin of a diameter of 9 mm. In order to smooth out the inequalities caused by machining, the pin surface which cooperates during the tribological test was ground on 240 gradation paper for 30 s.

### 2.2. Methodological Bases

Macrographs of the aluminum samples and polymer pins were taken with an Omnivision OV12A10 camera (Xiaomi, Haidian District, Beijing, China) while micrographs of the structure and morphology of the formed surface oxide layers were taken using a JEOL JSM-7100 TTL LV field emission scanning electron microscope (Jeol Ltd., Tokyo, Japan). The samples for the measurements were sputtered with gold. To calculate the diameters of the nanofibers and pores, ImageJ software 1.50i (LOCI, University of Wisconsin, Madison, US) was used. A DC GPR-25H30D GW Instek (IET labs, Inc., NY, US) power supply was used for hard anodizing process. The thickness of the oxide layers was measured with a Dualscope MP40 by Fischer (Helmut Fischer GmbH+Co.KG, Sindelfingen, Germany), using the eddy current method. Ten measurements were performed along the length of the sample and then the average value was calculated. Geometric parameters defining the structure were called SGP. SGP measurements of the oxide layers and the polymer pin were made by a Taylor Hobson Talysurf 2D pin profilometer (Taylor Hobson, Leicester, UK) with the accuracy of ±2%. Tribological measurements were performed on a T17 tester (Łukasiewicz Research Network—The Institute for Sustainable Technologies, Radom, Poland), pin-on-plate in reciprocating motion, at room temperature, at the humidity of 35% ± 5%, and using 0.5 MPa pressure at the average sliding speed velocity of 0.2 m/s in dry friction conditions. The tribological test was conducted for the sliding distance of 15 km. The sliding trace was 40 mm. A rectangular aluminum plate with the area of 0.1 dm^2^ was used as the sample and a pin of the diameter of 9 mm as the counter-sample. The average value of the coefficient of friction was calculated when the coefficient of friction change curve reached the rectilinear range. The wear quantity of the polymeric pin was studied using a WPA-60G (Radwag) analytical scale with the accuracy of ±0.1 mg, before and after each friction cycle.

## 3. Results and Discussion

**Figure 2:**
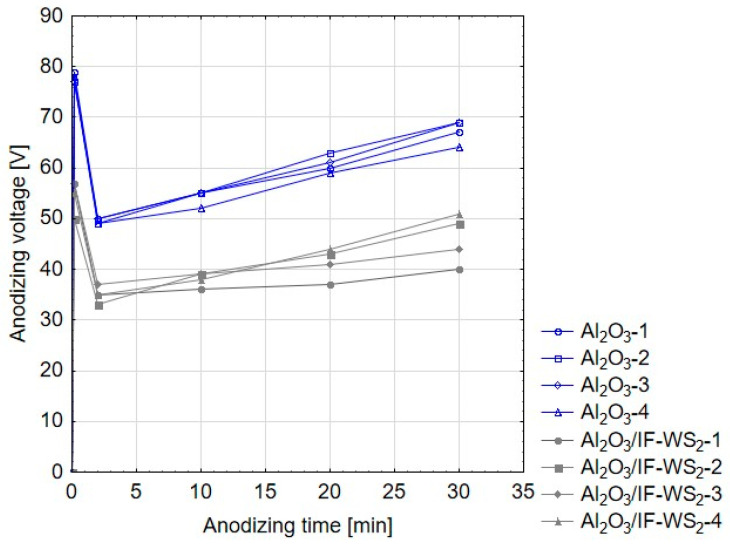
Anodizing voltage–time dependence for SAS and SAS electrolyte with admixture of IF-WS_2_.

**Figure 3:**
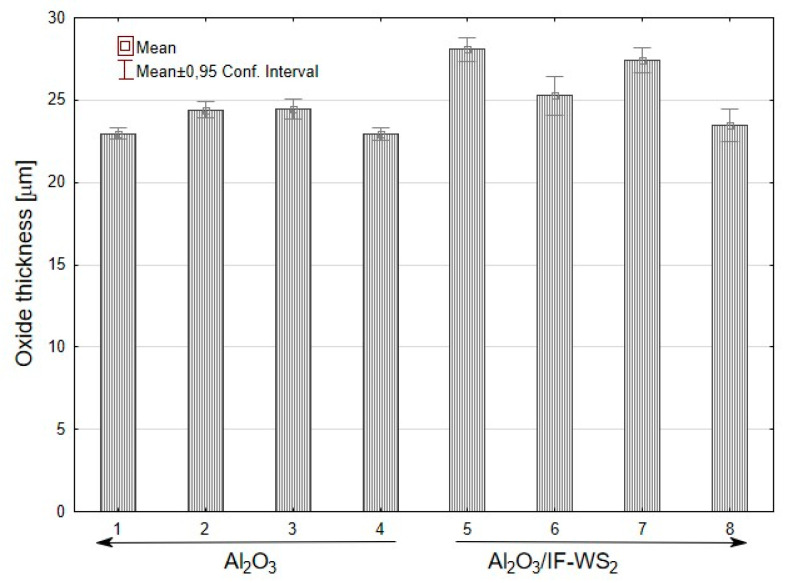
Thickness of the oxide layer on aluminium samples obtained in SAS and the SAS/IF-WS_2_ electrolyte.

Figure 2 shows the dependence of the anodizing voltage on time for the process carried out at the constant current density of 3 A/dm^2^. Anodization of the aluminum substrate in the SAS electrolyte and in the SAS electrolyte with the admixture of IF-WS_2_ exhibited a rapid increase and then a rapid decrease in voltage during the first seconds of the process. The relationship visible in the first seconds is related to dissolution of the compact native oxide film formed by exposure to air and distilled water used to remove residual acid after the second step of etching. At the minimum anodizing voltage, a barrier layer was created and the layer of aluminum oxide fibers began to increase. According to the graph, one can also observe certain differences between the SAS electrolyte and SAS electrolyte with the admixture of IF-WS_2_. During the oxidation process in the SAS electrolyte with dispersed IF-WS_2_ nanoparticles, lower values of voltage for all the tested samples compared to oxidation in the pure SAS electrolyte were observed. The observed increase in voltage is explained by the settling of IF-WS_2_ nanoparticles on the surfaces of the samples, which hindered uniform oxidation of the aluminum alloy. Therefore, during electrolysis the solution was mechanically mixed every 10 minutes to remove the settled IF-WS_2_ nanoparticles from the surface of the samples. While this activity was performed, an increase in voltage was observed. The temporary decrease in voltage observed during the short mixing of the solution may also cause the formation of slightly thicker layers that characterized the samples obtained in the SAS/IF-WS_2_ electrolyte (Figure 3).

The average values of the oxide layer thickness were in the range of 22.94–28.07 μm (Figure 3). The Al_2_O_3_/IF-WS_2_ samples showed a higher standard deviation. The larger differences in the oxide thickness on the sample surface were most likely caused by the uneven settling of the IF-WS_2_ nanopowders, which prevented an even exchange of oxygen-containing ions (O^2−^ or OH^−^) from the electrolyte and Al^3+^ ions through the oxide layer.

**Figure 4:**
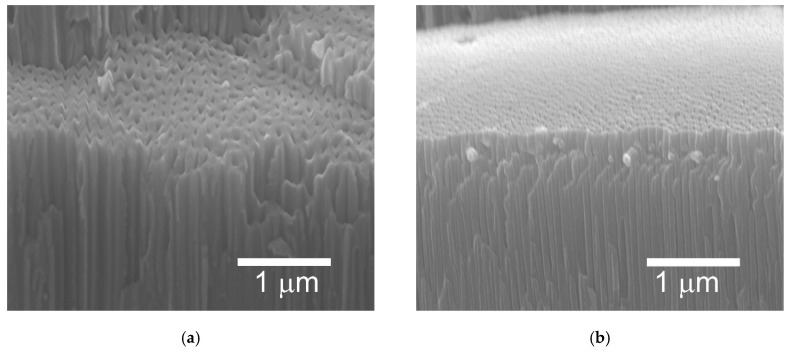
SEM micrographs of a fresh cross section of an oxide coating (**a**) Al_2_O_3_ and (**b**) Al_2_O_3_/IF-WS_2_.

**Figure 5:**
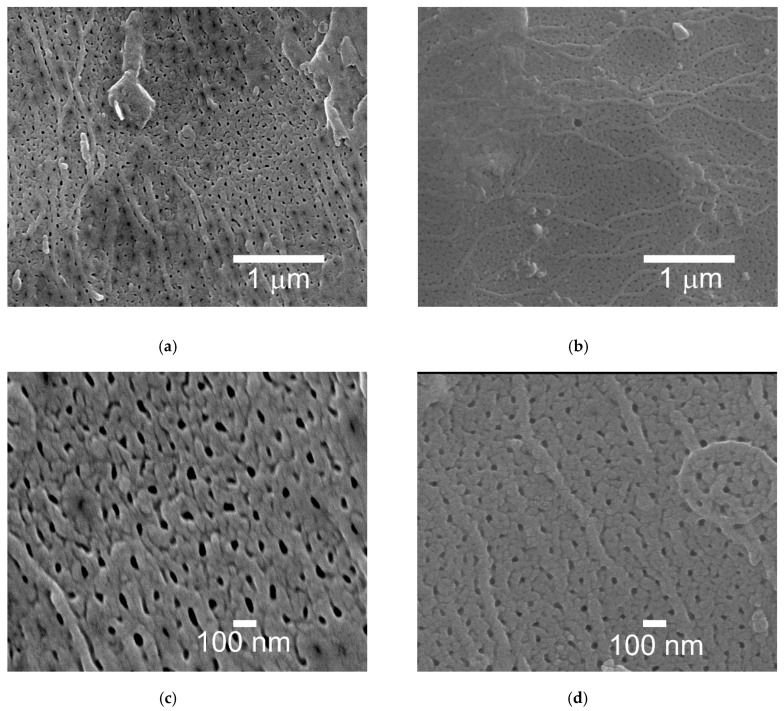
SEM micrographs of surface coating 20,000×: (**a**) Al_2_O_3_ and (**b**) Al_2_O_3_/IF-WS_2_ and 50,000×: (**c**) Al_2_O_3_ and (**d**) Al_2_O_3_/IF-WS_2_.

Figure 4 shows examples of SEM micrographs of cross sections of the oxide coatings. In Figure 4 the Al_2_O_3_ coatings (a) and the Al_2_O_3_/IF-WS_2_ coatings (b) are presented. In both cases the aluminum oxide nanofibers were visible. The mean value for the nanofibers of the Al_2_O_3_ coatings was 105 ± 11 nm, while for the nanofibers of the Al_2_O_3_/IF-WS_2_ coatings it was 67 ± 3 nm. The surfaces of the unmodified Al_2_O_3_ oxide layer are shown in Figure 5a,c, whereas the surfaces of the modified Al_2_O_3_ /IF-WS_2_ coating are shown in Figure 5b,d. Figure 5a,b were made with 20,000× magnification, while Figure 5c,d with 50,000× magnification. The mean size of the nanopores for the Al_2_O_3_ coatings was about 113 ± 50 nm, while for the Al_2_O_3_/IF-WS_2_ coating it was about 63 ± 35 nm.

**Figure 6:**
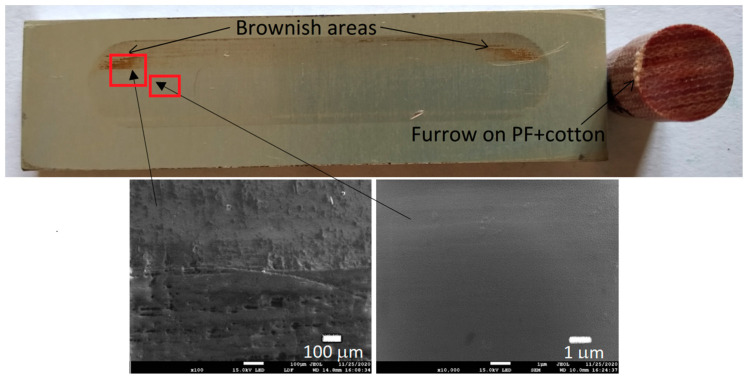
Photographs and SEM micrographs of Al_2_O_3_ tribological pairs after a tribological test with PF + cotton.

**Figure 7:**
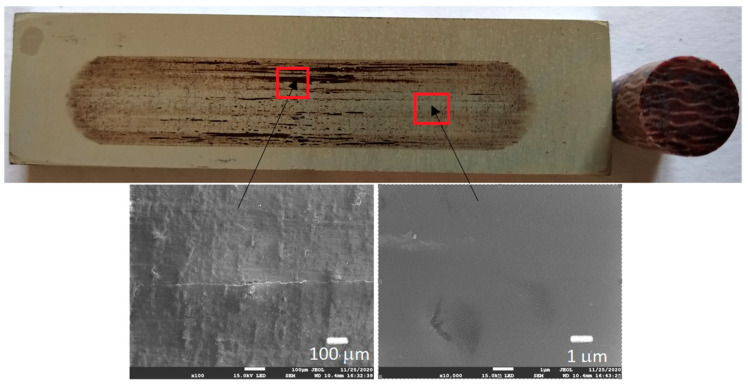
Photographs and SEM micrographs of Al_2_O_3_ tribological pairs after a tribological test with PF + jute.

**Figure 8:**
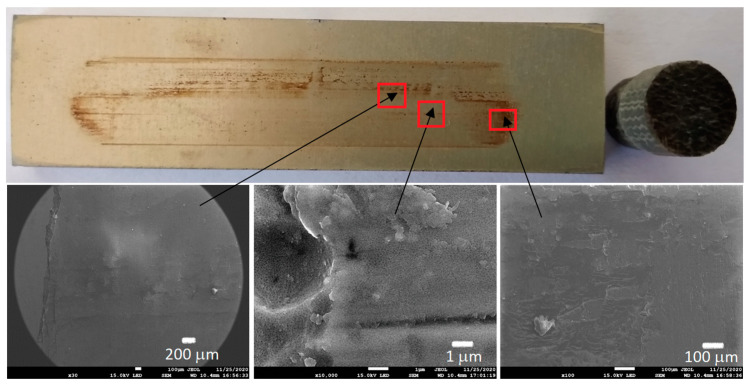
Photographs and SEM micrographs of Al_2_O_3_ tribological pairs after a tribological test with PF + cotton + MoS_2_.

**Figure 9:**
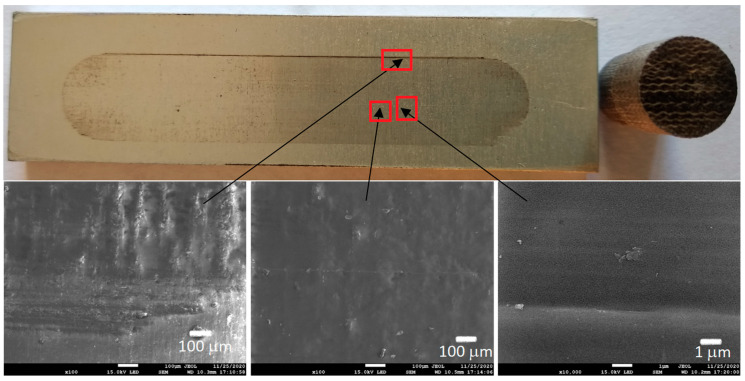
Photographs and SEM micrographs of Al_2_O_3_ tribological pairs after a tribological test with PF + cotton + C.

**Figure 10:**
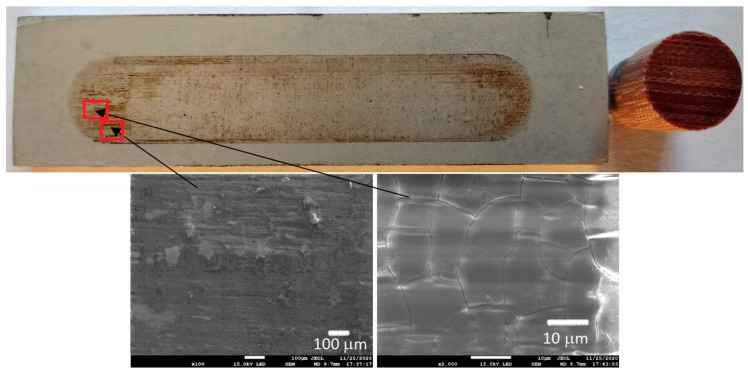
Photographs and SEM micrographs of Al_2_O_3_/IF-WS_2_ tribological pairs after a tribological test with PF + cotton.

**Figure 11:**
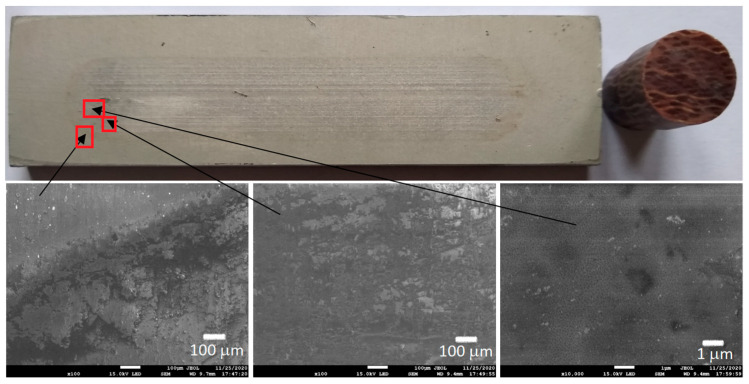
Photographs and SEM micrographs of Al_2_O_3_/IF-WS_2_ tribological pairs after a tribological test with PF + jute.

**Figure 12:**
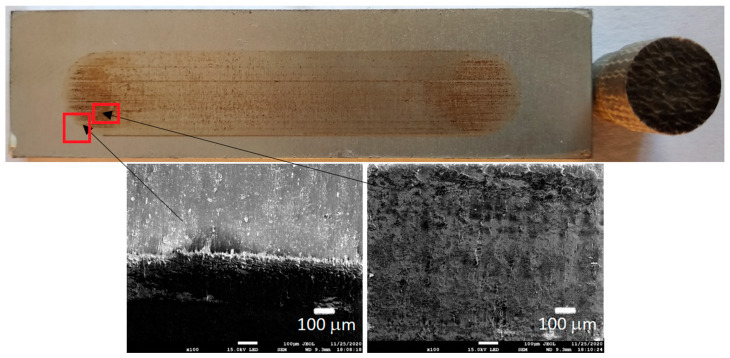
Photographs and SEM micrographs of Al_2_O_3_/IF-WS_2_ tribological pairs after a tribological test with PF + cotton + MoS_2_.

**Figure 13:**
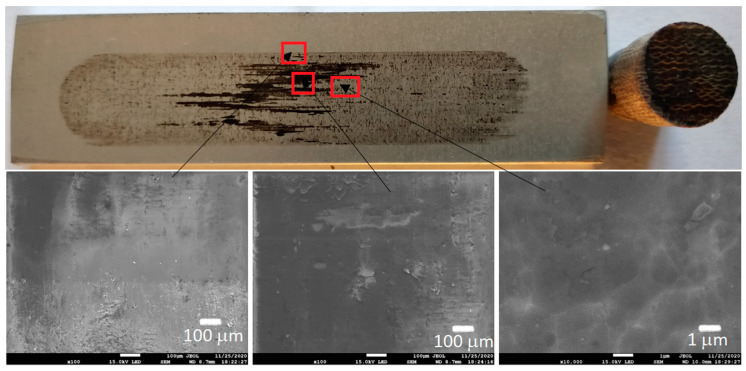
Photographs and SEM micrographs of Al_2_O_3_/IF-WS_2_ tribological pairs after a tribological test with PF + cotton + C.

**Figure 14:**
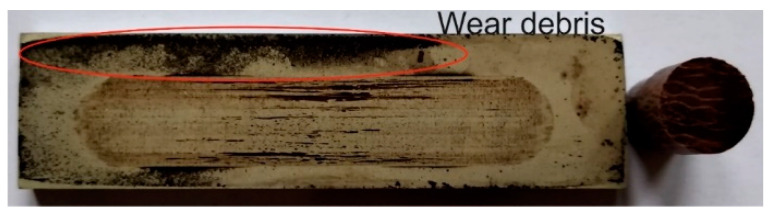
Al_2_O_3_ coating with visible wear debris from a pin of PF + jute.

**Figure 15:**
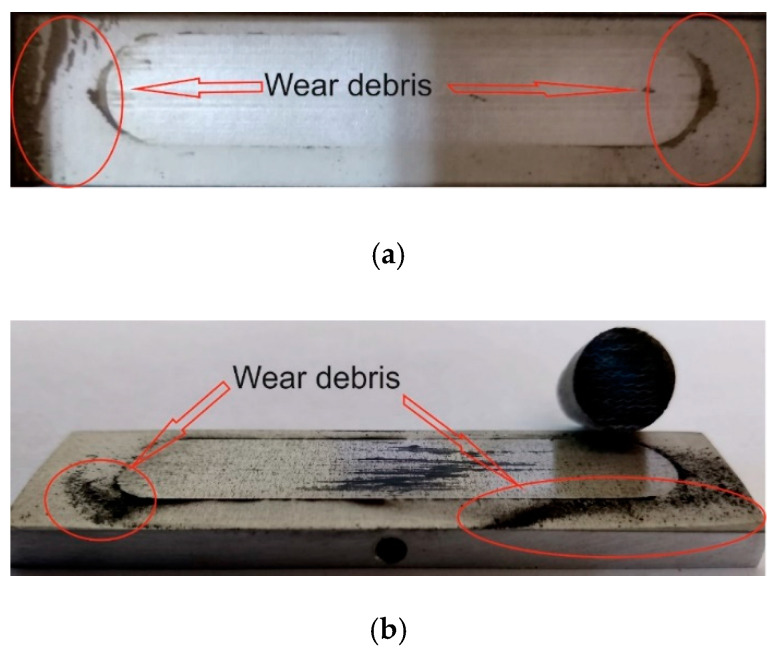
Samples with visible wear debris from a: (**a**) pin of PF + jute with visible bright wear debris on Al_2_O_3_/IF-WS_2_ and (**b**) pin of PF + cotton + C on Al_2_O_3_/IF-WS_2_.

Figure 6, Figure 7, Figure 8 and Figure 9 show the photographs and SEM micrographs of the Al_2_O_3_-PF tribological pairs after the tribological tests. The observations were made after removing the wear debris from the surface of the aluminum coatings. For the Al_2_O_3_-PF + cotton tribological pair (Figure 6), it can be seen that as a result of friction a transparent polymer film was formed on the surface of the aluminum oxide. Despite the even load applied to the pin, after tribological cooperation furrowing is visible on the PF + cotton composite pin surface. The width of this furrow corresponded to the width of the area on the polymer film created on the aluminum plate surface where the adhesion effect between the rubbing surfaces occurred. As a result, brownish areas at the ends of the polymer film were observed. A quite large area of brownish polymer film from the PF + jute was formed on the Al_2_O_3_ surface (Figure 7). A transparent polymer film with a brownish area was also formed by the Al_2_O_3_ coating-PF + cotton + MoS_2_ pair (Figure 8). For this tribological pair the shape of the film transferred to the Al_2_O_3_ coating also reflected the furrow area on the composite pin. A transparent, thin, smooth polymer tribofilm without visible traces of an adhesion effect was created during the tribological test between Al_2_O_3_ and PF + cotton + C (Figure 9). Figure 10, Figure 11, Figure 12 and Figure 13 show the photographs and SEM micrographs of the Al_2_O_3_/IF-WS_2_-PF tribological pairs after the tribological tests. A brownish film in the turning points of the friction path was visible on the surface of the Al_2_O_3_/IF-WS_2_ coating cooperating with the PF + cotton composite (Figure 10). A similar observation was noticed for the tribological pair Al_2_O_3_/IF-WS_2_ coating cooperating with PF filled with cotton and MoS_2_ (Figure 12). A transparent, thin, smooth adhesive film of the PF + jute was observed on the Al_2_O_3_/IF-WS_2_ surface coating (Figure 11) in contrast to the Al_2_O_3_ surface coating. In Figure 14 the Al_2_O_3_ coating with visible wear debris from the pin of PF + jute is shown. The bright color of the wear debris of PF + jute appeared after the tribological test with Al_2_O_3_/IF-WS_2_ (Figure 15a). In Figure 13 areas with a thicker layer of polymer film on the Al_2_O_3_/IF-WS_2_ coated plate were visible also in contrast to the Al_2_O_3_ surface coating. The wear debris from the pin of PF + cotton + C had a dark color (Figure 15b). As it resulted from the observation of the surface of the samples, areas with visible brownish or black traces of friction trace occurred mainly in the central part of the aluminum oxide sample, where the friction velocity was the highest, and near the turning points where the velocity was zero. The frictional conditions in these areas favored more intense heat generation and the formation of adhesive joints. Thus these local processes can play a major role in determining the course of wear, a similar observation was also reported elsewhere [38]. The tribological films on the surfaces of aluminum oxide coatings were the result of abrasive wear of the polymer pins. In the turning points, the adhesive wear of the polymer pins takes place. A strong junction between rubbing asperities of surfaces is formed in the micro-areas. When a joint is broken, the dynamic effect in detaching of the fragment of the polymer pin and formation of the transfer film on the friction surfaces or/and the loose wear debris is observed. This can be attributed to the transfer layer formation based on adhesion [39]. In the vast majority of tribological associations, not only one type of wear occurs, and usually there are several types of wear increasing simultaneously. 

**Figure 16:**
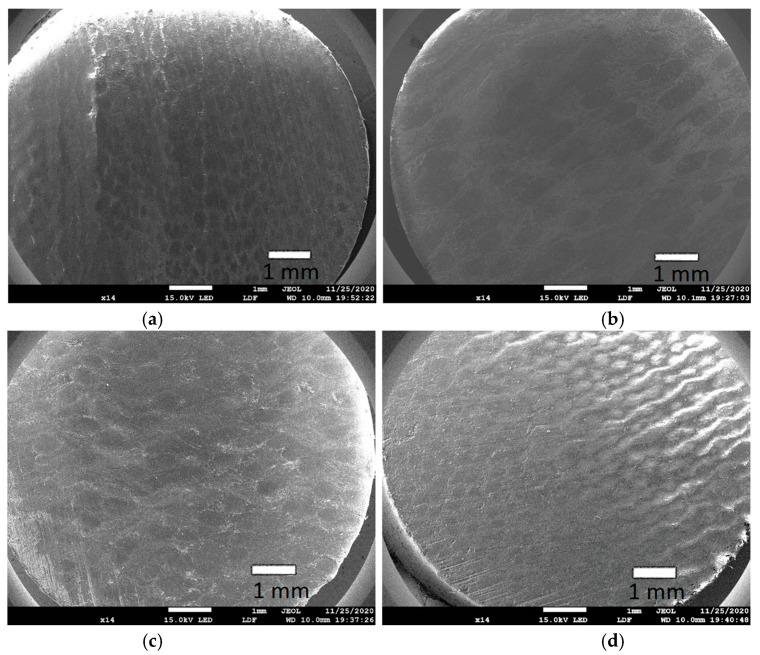
SEM micrographs of pins: (**a**) PF + cotton (**b**) PF + jute (**c**) PF + cotton + MoS_2_, and (**d**) PF + cotton + C.

In Figure 16a–d the SEM micrographs of pins of PF composites are shown. The cotton fibers were visible in Figure 16a,c,d, and jute fibers in Figure 16b.

**Figure 17:**
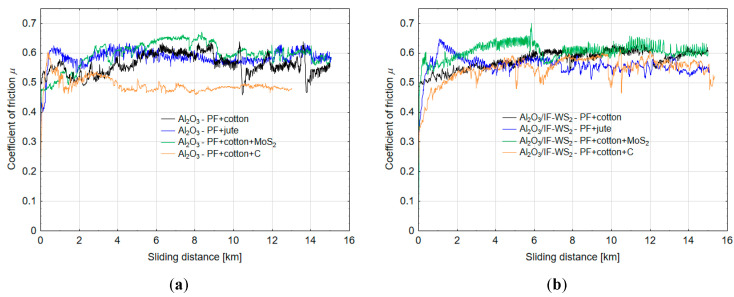
Coefficient of friction vs. sliding distance for tribological pairs of: (**a**) Al_2_O_3_–PF composites and (**b**) Al_2_O_3_/IF-WS_2_-PF composites.

In Figure 17a,b graphs of the coefficient of friction vs. sliding distance for the pairs with Al_2_O_3_ and Al_2_O_3_/IF-WS_2_ are presented, respectively. The sudden increases and decreases in the value of the coefficient of friction after 10 km for the Al_2_O_3_ coating cooperating with PF + cotton (black graph in Figure 17a) were caused by adhesive tacking between the cooperating elements. Those areas on the surface coating were visible as brownish places in Figure 6. A similar magnitude of disturbance in the course of the coefficient of friction is shown for the Al_2_O_3_/IF-WS_2_ coating cooperating with the PF + cotton + C (orange graph in Figure 17b). The sudden decreases in friction could be the effect of the appearance of the loose wear debris of the polymer between the two surfaces. The nature of changes in the coefficient of friction were comparable for tribological pairs with both the Al_2_O_3_ and Al_2_O_3_/IF-WS_2_ oxide layers cooperating with individual polymer composites. However, it can be noticed that the course of changes in the value of the friction coefficient as a function of the friction path was more stable for the case of the Al_2_O_3_/IF-WS_2_ oxide layer.

**Figure 18:**
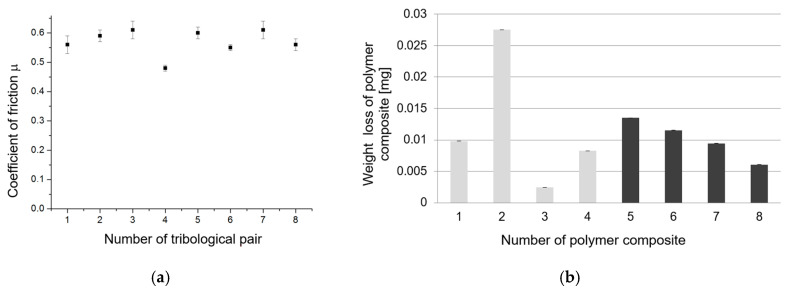
(**a**) Average values of coefficient of friction for tested tribological pairs and (**b**) weight loss of polymer composites.

In Figure 18a the average values of the coefficient of friction for the studied tribological pairs are shown. For the Al_2_O_3_ coatings the following values were calculated: μ_1_ = 0.56 ± 0.03 (PF + cotton), μ_2_ = 0.59 ± 0.02 (PF + jute), μ_3_ = 0.61 ± 0.03 (PF +cotton + MoS_2_), and μ_4_ = 0.48 ± 0.01 (PF + cotton + C). For the Al_2_O_3_/IF-WS_2_ coating the values of friction coefficient were as follows: μ_5_ = 0.60 ± 0.02 (PF + cotton), μ_6_ = 0.55 ± 0.01 (PF + jute), μ_7_ = 0.61 ± 0.03 (PF + cotton + MoS_2_), and μ_8_ = 0.56 ± 0.02 (PF + cotton + C). The determined values of the friction coefficient are comparable to those reported in the literature for amorphous thermoplastic polymers ((polycarbonate—PC, polyamide-imide—PAI, and polyetherimide—PEI) [29], or polyoxymethylene (POM) [38] cooperating with steel. The combinations of the Al_2_O_3_ coating with polymers with the addition of cotton showed little coverage of the coatings with transparent polymer layers (Figure 6, Figure 7 and Figure 9). By far the best polymer lubrication efficiency was obtained by the combination of the Al_2_O_3_ coatings with the PF + cotton + C composite (Figure 9). Good cooperation of the triboelements was characterized by the lowest value of the coefficient of friction from all the studied tribological pairs (Figure 18a). The PF filled with cotton +MoS_2_ combined with the oxide layer showed the highest values of the coefficient of friction from all the examined pairs (Figure 18a). The combination of the Al_2_O_3_ coating with the PF + jute showed significant run-in of the sliding distance, which was the effect of transferring the polymer material from the pin to the surface of the coating and then sticking these particles to the surface of the pin. The dark colors on the friction surfaces and significant coverage of the plate surface indicate a higher value of friction force, which is confirmed by Figure 18a. Unfortunately, the interaction of this sliding pair also resulted in the highest polymer wear value (Figure 18b), mainly due to the fact that the polymer was dusted heavily during the tribological test (Figure 14). The addition of lubricants to the PF caused the lowest polymer wear in the case of PF + cotton + MoS_2_ and the lowest value of the coefficient of friction for PF + cotton + C. In the case of the association of Al_2_O_3_/IF-WS_2_ coatings with PF composites, a similar behavior was observed as for the associations of these composites with the Al_2_O_3_ layer. The combination of Al_2_O_3_/IF-WS_2_ with PF + cotton + C was also characterized by the lowest value of the coefficient of friction (Figure 18a). Despite the fact that for this tribological pair a polymer film with significant adhesive tacking and visible polymer dusting was observed (Figure 15b), this tribological pair was also characterized by the minimal wear value of the polymer pin (Figure 18b).

Figure 18a shows that the lowest values of the coefficient of friction were obtained in the case of the PF + cotton + C, cooperating with the aluminum oxide layer produced by both oxidation methods. However, the differences between the values of the coefficient of friction determined for the friction pairs with aluminum samples with different oxide layers were small. Thus, it can be concluded that in the frictional connections between the hard oxide layer and the polymer composite, the properties and chemical composition of the soft material, i.e., the polymer composite, whose components could act as a solid lubricant, are of greater importance for the course of the friction process than the type of aluminum surface layer modification. In Figure 18b a bar graph of the weight loss of the polymer composites is presented. The highest value of weight loss equal to 0.027 mg was noticed for the pin made of PF + jute after the tribological test with Al_2_O_3_. The lowest one equaled 0.002 mg was for the pin made of PF + cotton + MoS_2_ after the tribological test with the Al_2_O_3_ coatings. Quite good results were obtained for the PF + cotton + C cooperating with both types of aluminium oxide layers. For these friction pairs the coefficient of friction also had the lowest values (Figure 18a) and the aluminium oxide surface, after the friction process, was smooth, without a visible adhesive wear effect or only some dark wear debris gathered on the surface of the aluminium oxide layer prepared using the SAS electrolyte with an admixture of IF-WS_2_ (Figure 13).

**Figure 19:**
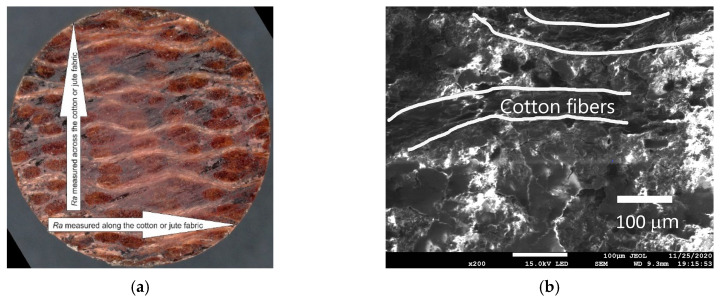
(**a**) Direction of roughness (Ra) measurements on the surface of the polymeric composites pin and (**b**) SEM micrograph of the PF+cotton+MoS_2_ pin.

The average value of the surface roughness (Ra) of the polymer composite pins before friction was 3.1 ± 0.3 μm. Figure 19a shows the measurement directions of surface roughness (Ra) of the polymer composite sample. The roughness was measured along and across the arrangement of fibers embedded in the phenol-formaldehyde resin. Figure 19b shows the SEM micrograph of PF+cotton+MoS_2_ pin with marked fibers.

**Figure 20:**
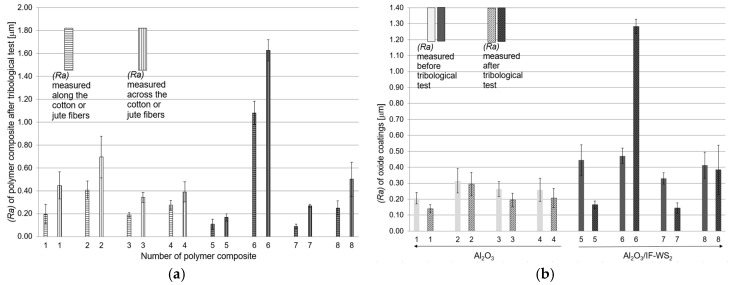
Roughness values (*Ra*) for: (**a**) the surface of the polymer composite pin after the tribological test and (**b**) aluminum oxide surfaces obtained in SAS and SAS/IF-WS_2_ electrolyte before and after the tribological test.

Figure 20a presents the roughness values (Ra) of the polymer composite surfaces after tribological tests. The polymer pin was positioned so that its fibers were along the direction of friction. The values of roughness measured across the cotton or jute fibers was higher than those measured along the fibers. The surface roughness measurements of the Al_2_O_3_ and Al_2_O_3_/IF-WS_2_ oxide layer were made in the direction perpendicular to the direction of friction movement. Figure 20b shows the roughness values (Ra) of the Al_2_O_3_ and Al_2_O_3_/IF-WS_2_ surfaces. The (Ra) values were in the range of 0.19–0.31 μm for the Al_2_O_3_ layers and 0.33–0.47 μm for the Al_2_O_3_/IF-WS_2_ layers. It is noticeable that for the layers obtained in the electrolyte with the IF-WS_2_ admixture, the coatings had slightly higher surface roughness compared to the values determined for the surface of the oxide coatings obtained in the SAS electrolyte (the bar chart before the tribological test in Figure 20b). In Figure 20b it is also observed that the surface roughness values after the tribological tests were lower than the values before the tribological tests; one exception was the Al_2_O_3_/IF-WS_2_ surface coating after the tribological test with PF + jute. During the friction test the softer polymer film filled the unevenness of the oxide coatings, hence in almost all the cases the oxide surface was smoothed. For tribological pair number 6 (Figure 11), the highest roughness values for both the Al_2_O_3_/IF-WS_2_ oxide coating and the composite of PF + jute were recorded after the tribological test. In this case, deep abrasive scratches were formed on the Al_2_O_3_/IF-WS_2_ oxide layer. Parallel furrows formed on the pin surface of PF + jute correspond to the locations of the scratches of Al_2_O_3_/IF-WS_2_. Hence the high roughness values on both the friction materials of pair number 6 were observed.

The kind of formed tribofilm and dependence of coefficient of friction vs. time show that the results most likely were affected by the type of polymer composite filler (cotton, jute, cotton + MoS_2_, and cotton + C) and the microgeometry of the polymeric pin surface, which is also mentioned by [40]. The softer polymer filled the microdefects of the Al_2_O_3_ and Al_2_O_3_/IF-WS_2_ surface coatings and in almost all the cases the oxide layer surface was smoothed by the tribofilm created from the polymer composite. Consequently, the pin of the polymer composite was rubbing against the polymer film on the Al_2_O_3_ and Al_2_O_3_/IF-WS_2_ oxide layer and not against the oxide itself. This research delivered information about the association of the commercially available polymer and obtained oxide coatings. It is worth looking at the tribological and frictional properties of innovative surface solutions at the level of basic research, which is in line with the view shown in [29,41]. Despite the progress made, a number of key questions, however, remain unanswered.

## 4. Conclusions

The conducted tests showed the durability of oxide coatings in combinations with polymer composites made on the matrix of phenol-formaldehyde resin, filled with cotton fiber, jute, and additionally with graphite or molybdenum disulphide. Addition of IF-WS_2_ nanopowder to the SAS electrolyte increased the roughness of the aluminum coating. The tribological tests showed that on the 15 km distance, under the load of 0.5 MPa, the tested combinations of friction pair elements show a stable course of changes in the coefficient of friction value, a little better for oxide layers modified by IF-WS_2_ nanoparticles. Taking into account the standard deviations, comparable values of the coefficient of friction for pairs associated with the Al_2_O_3_ layers obtained in the SAS and SAS/IF-WS_2_ electrolyte were obtained, except for pair 4. Nevertheless, for all the tested friction pairs, a relatively high coefficient of friction was recorded, amounting to over µ = 0.48, which does not allow them to be defined as proposals for self-lubricating sliding contacts, however maybe applicable quite well under certain practical conditions. The lowest values of the coefficient of friction were characteristic for the oxide layers made on aluminum in contact with the polymer composite filled with cotton fiber and graphite. In the case of this combination, good surface condition of the aluminum layer after the friction process was observed, which was characterized by low surface roughness and no visible traces of adhesive polymer build-up. Further research is needed to modify the polymer composites, for example soaking the fibers incorporated into the polymer in oil in order to obtain combinations with a low coefficient of friction.

## Figures and Tables

**Table 1:** Names and descriptions of studied samples.

Number of Tribological Pair	Oxide Layer-Sample	Polymer Composite
1	Al_2_O_3_-1	PF + cotton
2	Al_2_O_3_-2	PF + jute
3	Al_2_O_3_-3	PF + cotton + MoS_2_
4	Al_2_O_3_-4	PF + cotton + C
5	Al_2_O_3_/IF-WS_2_-1	PF + cotton
6	Al_2_O_3_/IF-WS_2_-2	PF + jute
7	Al_2_O_3_/IF-WS_2_-3	PF + cotton + MoS_2_
8	Al_2_O_3_/IF-WS_2_-4	PF + cotton + C
